# A Landmark-Guided Pericardiocentesis Simulator for Resource-Limited Procedural Training: A Development Informed by Prior Techniques

**DOI:** 10.7759/cureus.91234

**Published:** 2025-08-29

**Authors:** Brian F Quach, Alexander S Hayden, Shayne J Welsh, Andrew Eyre

**Affiliations:** 1 School of Medicine, Frank H. Netter MD School of Medicine at Quinnipiac University, North Haven, USA; 2 Emergency Medicine, Brigham & Women’s Hospital, Harvard Medical School, Boston, USA

**Keywords:** accessible medical simulation, emergency medicine procedures, emergency pericardiocentesis, landmark-guided pericardiocentesis, low-income resource-limited countries, medicine in resource limited areas, resource-limited hospital, resource limited setting, rural emergency medicine, teaching in emergency medicine

## Abstract

Pericardial tamponade is defined as a medical emergency where the pericardium of the heart fills with fluid, compressing and preventing it from pumping blood efficiently. If left untreated, progressive hemodynamic instability and cardiac arrest would occur. The management of this clinical condition necessitates a pericardiocentesis, which is the aspiration of fluid from the pericardial sac. For high-acuity, low-occurrence scenarios like this, medical simulation provides a psychologically safe environment to practice advanced emergency procedures such as pericardiocentesis. However, due to financial and supply chain constraints, it may be difficult to ensure adequate access to training simulators for clinicians practicing in remote and rural areas. To address this issue and to support skill acquisition for clinicians practicing in these areas, we designed a cost-effective and reusable pericardiocentesis trainer, building upon prior methods using ballistic gels as the medium for model making. The simulator is designed to allow adequate practice of both landmark-guided and ultrasound-guided pericardiocentesis from the three main approaches (substernal, apical, and parasternal).

## Introduction

In emergency settings, timely recognition of acute cardiac tamponade heavily relies on identification of signs and symptoms using traditional physical exam maneuvers and ultrasound imaging [1]. Typical symptoms may include, but are not limited to, hypotension due to decreased venous preload, muffled heart sounds, and distended jugular veins signifying impeded venous inflow. In these circumstances, fluid such as blood can accumulate in the pericardial space of the heart, and drainage can become impeded due to underlying pathophysiology. One example is the effects of chronic pericardial effusions. These effusions may convert to tamponade in patients with poorly controlled pathologies such as infection, uremia, malignancy, and post-myocardial infarction syndrome (Dressler’s syndrome), requiring procedural intervention [1,2]. Pericardiocentesis for treatment of tamponade is a high-risk procedure that is performed in modern medicine. With the introduction of ultrasound sonography, which can triage acuity of the tamponade, medical providers may potentially lose their psychomotor skills in performing landmark-guided pericardiocentesis when an ultrasound is unavailable [2].

Medical simulation is an educational adjunct that can allow medical providers to practice invasive procedures like pericardiocentesis in a psychologically safe setting. However, in resource-limited settings, challenges such as cost and supply chain logistics may limit access to simulators. With the goal of improving access to training opportunities, we designed a simulated task trainer for practicing blind pericardiocentesis with ballistics gelatin, building on prior techniques presented in the literature. This trainer includes anatomical landmarks that closely resemble those found on real human torsos for the blind approach and is echogenic to allow users to practice with ultrasound guidance if applicable.

Objective

We sought to create a cost-effective, easily reproducible model for training residents and other qualified healthcare professionals to perform pericardiocentesis based on prior techniques [1-5]; a procedure that, although infrequently required, is a life-saving intervention that can significantly alter the prognosis of a patient with acute cardiac tamponade. Given the high cost of some models, we considered it important to find a way to ensure educational centers, regardless of funding, could create an adequate training model [5-7]. Furthermore, we aimed to create a model that would be cheaply reusable with construction materials that could be easily purchased at a local store or ordered and delivered to a resource-limited setting [4,7-10]. Given the infrequently needed yet potentially critical nature of this procedure, allowing medical practitioners to continuously practice is something we feel is important [1,2]. 

## Technical report

For our pericardiocentesis trainer design, we utilized a combination of cost-effective and repurposed items (Table 1). A life-size skeleton model was utilized for the chest by using an oscillating saw to remove the spine, allowing just the rib cage to be placed into a plastic mold of a male chest. We then used a knife to cut the ballistic gel into small cubes to aid in the melting process. The cubes were placed into slow cookers, and after approximately two hours, they were melted down sufficiently for pouring purposes. The melted ballistic gel was then poured into the mold to ensure the rib cage did not shift during the hardening process to maintain anatomical accuracy. Meticulous placement of the rib cage is paramount for creating a realistic model that could have proper landmarks for performing pericardiocentesis both with and without ultrasound guidance. Failure to perform this step correctly may compromise the anatomical accuracy of the simulator, affecting the way providers perform the procedure on the simulator.

**Table 1:** Total Cost of Materials USD - United States Dollar Note: Items are typically bought in bulk and individual units are used to create the model. Over time the costs of components are subject to change in value. Total cost of the design may vary depending on the manufacturer, retail price, and quantity of items. The specific brand of components listed above are not essential in creation but simply what we used to design the simulator. Prices are reflective of USD as of April 2025.

Model Components	Cost (USD)	Amazon Standard Identification Number (ASIN)
Male mannequin torso mold	$38.75	B0842YBWK8
Rib Cage and Spine	$68.97	B091G2B71Y
Ballistics gel	$113.98	B0B9YNSFYP
Translucent Resin Dye	$9.99	B09JKZJGJB
Ping pong balls	$0.08 (for one)	B0DSJ3X37Y
Ultrasound probe cover	$1.92 (for one)	B0B56NGQXY
Enteral feed bag	$13.99	B0DHPK2X8Q
Total Cost of Design	$247.68	

Ballistic gel was chosen as the medium for our model due to its echogenicity under ultrasound imaging, if desired. Ballistic gel is also noted to be more life-like in comparison to gel wax, which we opted not to utilize as we were attempting to achieve maximum realism. Pouring of the melted ballistic gel was conducted in two stages directly into the chest mold to ensure anatomical accuracy by correcting any shifting contents.

Between pours, the enteral bag tubing was connected to the pericardial sac that we created out of an ultrasound probe cover. We decided that enteral bags were a cheap and effective reservoir for fluid directly to our simulated ultrasound probe cover pericardium. In addition to being a simulated pericardium, the ultrasound probe cover has the added benefit of being easily penetrable and can be utilized multiple times. This tubing carried water to simulate blood in the pericardial space that the trainee can aspirate. Tubing was covered in ballistic gel between pours in an effort to have it stick better to the model. Fluid-filled ping pong balls were placed within the ultrasound probe cover to simulate the ventricular wall appearance under ultrasonography [8]. 

**Figure 1:**
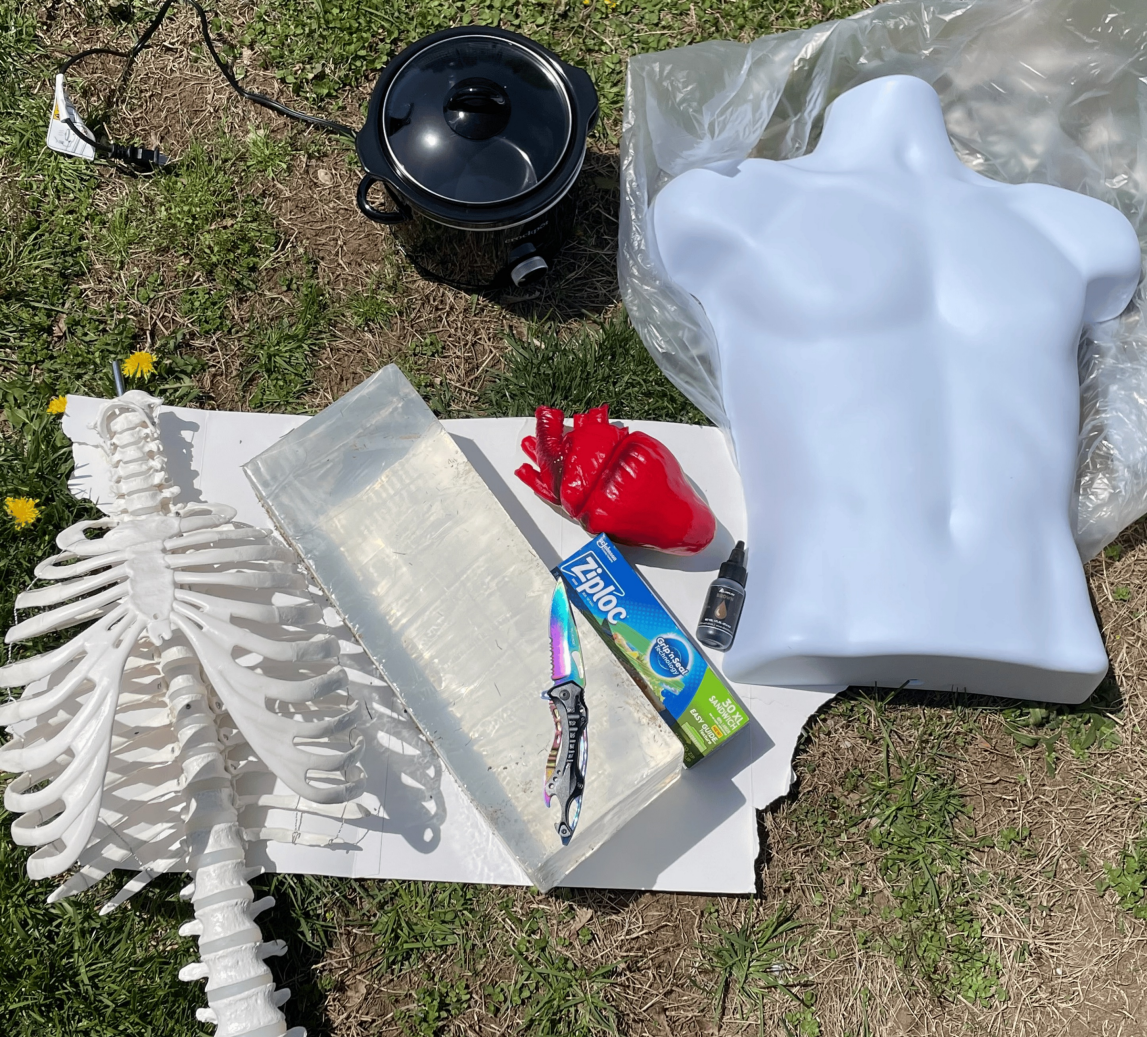
Component Materials For Model Making Please note that, due to poor echogenicity, the foam heart shown in this photo was not used in the final model. The heart was instead replaced by ping pong balls and a plastic ultrasound probe cover not shown here.

Once the gel was completely hardened, we removed the clamps on the enteral bag tubing and allowed water to flow into the pericardial sac, and the model was now complete and ready for testing. Figure 1 shows the component materials used for making the model.

Approximately 250 milliliters (mLs) of fluid were added to our pericardial sac. Though volume may vary to achieve adequate pericardial sac decompression in humans, benign pericardial effusion is typically 15-50mL in volume. If needed for the pericardial fluid, different color dyes (depending on the type of simulated fluid desired) could be added easily as the enteral bag remained on the outside of the model to the side for easy access.

Results

**Figure 2:**
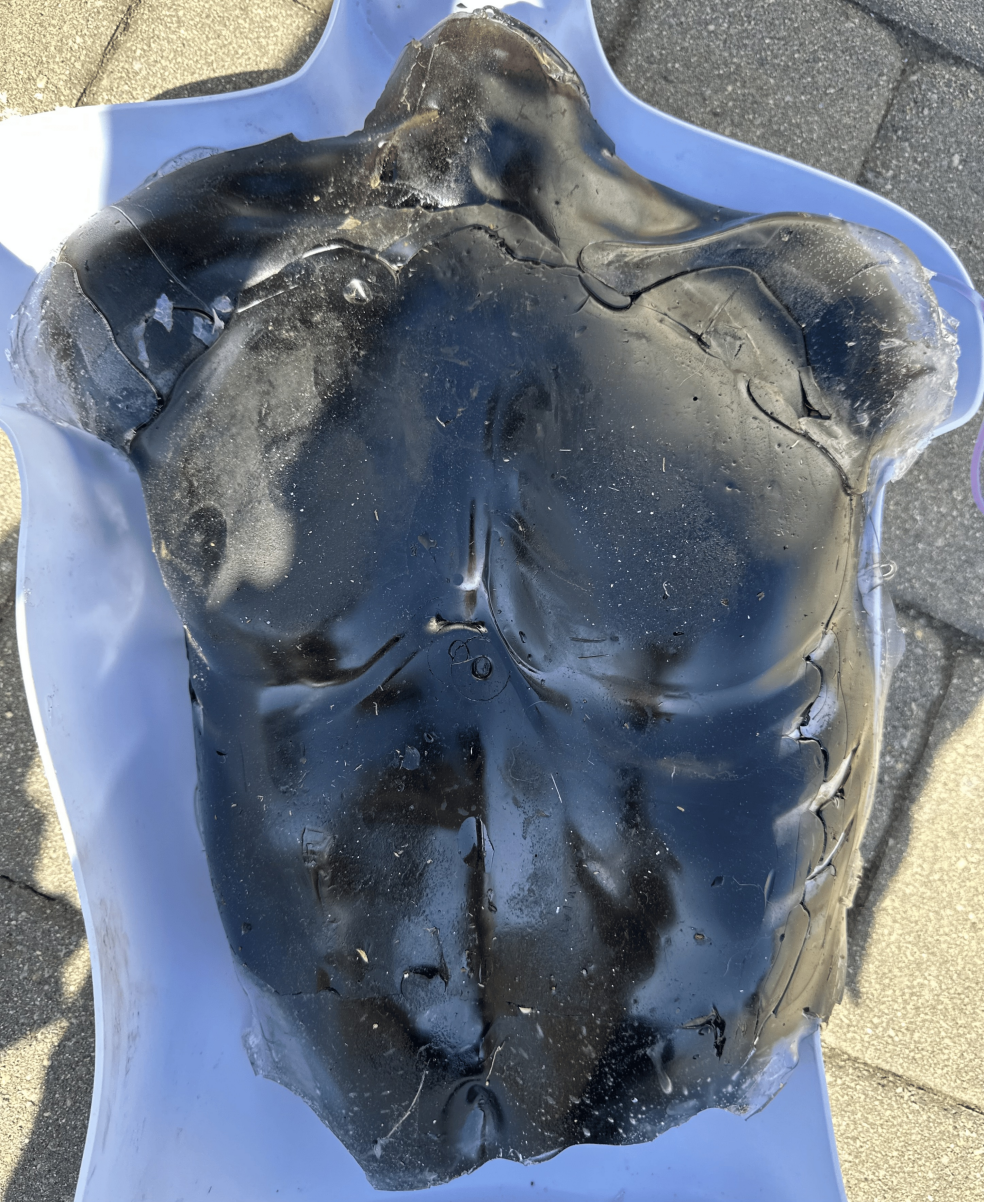
Completed Model

**Figure 3:**
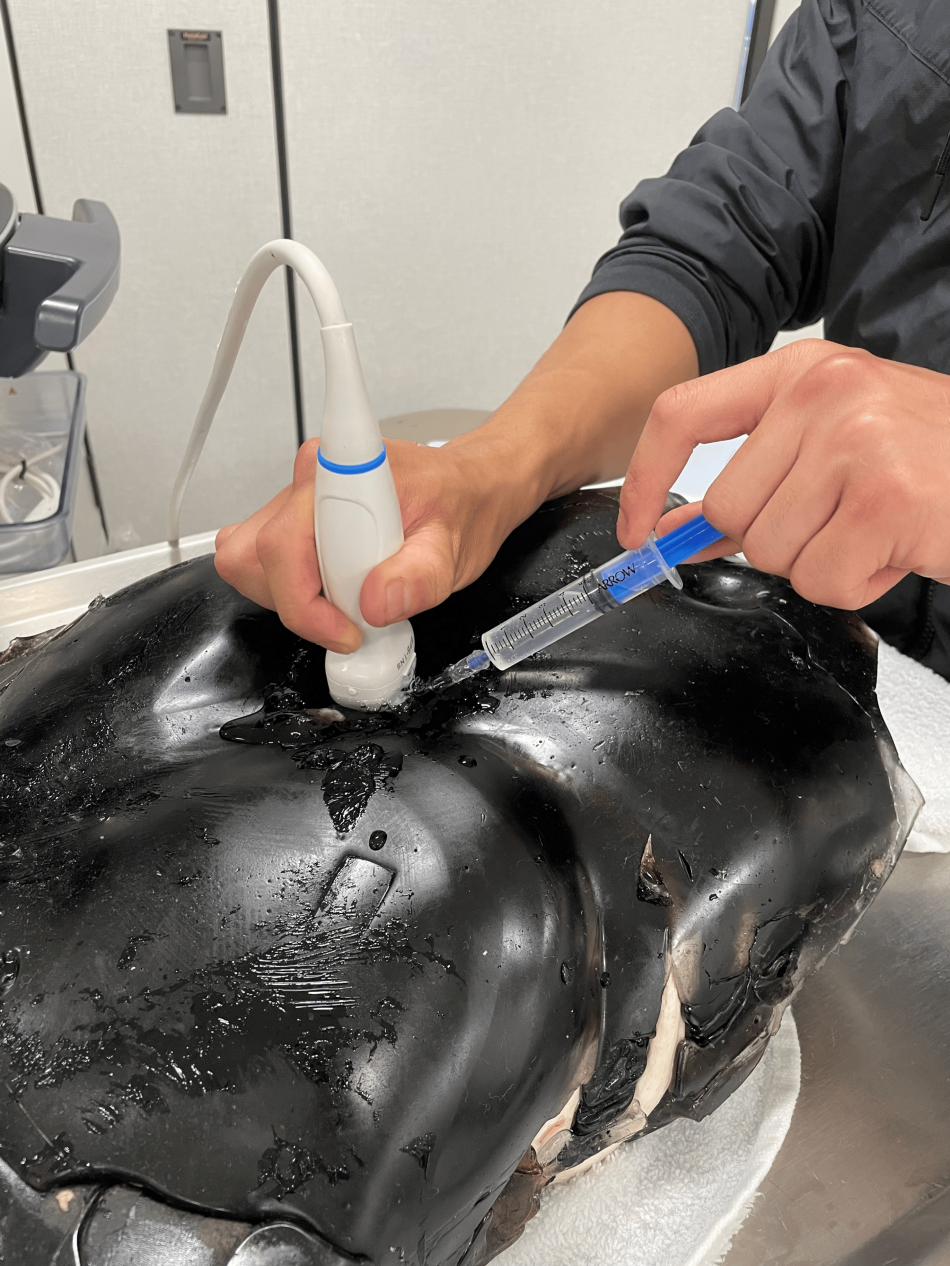
Model In Use With Ultrasound Guidance

We were able to create a sustainable and reusable model for pericardiocentesis training utilizing cost-effective and repurposed materials. The monetary cost for the construction of this model is US$247.68. This versatile model allows trainees to practice the technical skills required to utilize ultrasound to perform a pericardiocentesis. The inflatable pericardial sac made from an ultrasound probe cover allows the users to replace the drained-off fluid via the enteral feed bag that is embedded into the gel and directly into the simulated pericardium. Users may also practice performing the procedure utilizing landmarks such as the xiphoid process and ribs. Future iterations of this model may include a more self-sealing gel, as there was some leakage in prior needle sites. Figure 2 shows the completed model, and Figure 3 depicts the model in use with ultrasound guidance.

## Discussion

The importance of pericardiocentesis training in the skillset of medical providers across all areas is paramount. Financially underserved emergency care clinics may not have access to the currently available commercial models, and this was a major reason we pursued the creation of a cost-effective model. 

The majority of the cost of this model came from the ballistic gel. The slow cooker is also a costly component, though this expense can be avoided if one is already available, as in our case. It is possible to use even cheaper materials, such as a silicone filler typically used for creating watertight seals. It is echogenic on ultrasound as well, and in some studies, this property is taken advantage of. For example, in cases of silicone breast implant leakage, a “snowstorm” effect on ultrasonography is noted when attempting to find and diagnose a leak [11]. This material, however, may be more difficult to work with depending on the formulation utilized.

The advantages of this model are that it allows practitioners to hone a skill that, although infrequently utilized, can be a life-saving measure [1]. Also, it is possible to perform the procedure on this model either ultrasound-guided or landmark-guided from common approaches, the subxiphoid, apical, and parasternal. With the advent of echocardiography-guided pericardiocentesis in the 1970s, landmark (blind) procedures have been less prevalent due to the much lower rates of morbidity and mortality [6,12,13]. As the blind approaches are no longer utilized as often, it is important for medical providers to practice this method safely in the event ultrasound is not available. Given the cheap cost of the materials and high accessibility as well, this model can provide a trainer for remote institutions that lack the resources of larger, well-established, and funded academic centers. Ease of assembly in a fairly minimal amount of time, with very little required technical skill, proves beneficial in our model. 

Our model could impart more realism by adding solid and hollow structures to simulate organs such as the stomach, lungs, liver, intestines, spleen, and great vessels. This would require a more meticulous setup of the chest and upper abdominal cavity in order to maintain anatomic fidelity. With the introduction of these additional structures, our model could provide a more realistic experience to trainees in the process of learning or maintaining this skill by increasing the risk of simulated complications. Granted, with ultrasound technology and better training, these complications are increasingly rare, with about a 1-3% risk of any morbidity and less than 1% mortality [12].

Future studies

In order to determine the overall effectiveness of this model and its simulative benefits, we aim to recruit emergency medicine residents at a major academic institution to utilize our model and trial the different approaches for performing pericardiocentesis. Post simulation, we will provide them with a survey utilizing questions on a Likert scale to obtain data on their experience. This will allow us to discover the full role this model could play in the training of resident physicians and its effectiveness in learning the procedure and building confidence so that they may feel prepared to deploy this skill in a real-life high-stress situation. 

Limitations of the simulator

Firstly, being in the controlled, low-stress environment of a simulator compromises realism. Given that there are no stakes here, no real patients to be concerned with, and no high-octane, stressful, time-limited window to perform the procedure on a decompensating patient, realism is hard to achieve. Despite the most creative, detailed scripting and time limitations set for the trainee, it is impossible to truly recreate the feeling of being under pressure to save a life. However, a future iteration of this model could have more of an interactive scenario, perhaps with a type of artificial intelligence voice control to give the trainee a feeling of interviewing a patient who was in a trauma, such as a penetrating chest wound, who goes from stable to decompensating due to tamponade. This could potentially simulate the more dire nature by hearing the frantic patient go from conscious to unconscious. However, our model is created to limit cost, and this may prove to be not financially feasible. Although given the widespread research and recent explosion into the AI realm, perhaps it will become more financially available in the future. Speaking further on realism, a cadaver was utilized in other simulation models cited in literature, but given the cost and location of accessible cadavers [3,7], this can prove to be prohibitive depending on the simulation center and location of trainees. 

Secondly, there is a lack of other organ systems and body cavities that could potentially be injured in the process of practicing. Needle decompression of the pericardium is the goal of pericardiocentesis; however, there are massive complications that, while very rare these days with more advanced techniques such as ultrasound and improved landmark approaches, could potentially arise. Risks of arrhythmia, pericardial thrombus, vaso-vagal response, pneumothorax, cardiac trauma requiring surgical repair, great vessel trauma, and abdominal visceral trauma are nonexistent in this model. A future model could potentially have more comprehensive internal organ structures placed to create added risk and realism to the procedure for trainees who are practicing their technique. Rubber tubing filled with pockets of fluid to stimulate blood vessels or hollow tubing to simulate the stomach or intestine could also be considered.

Finally, we have not had any medical providers test it yet. Other than our own use in the simulation lab, it has remained untested. In our further studies, we may seek to have emergency medicine residents utilize the model to ascertain its benefit in training.

## Conclusions

Utilizing easily obtainable materials and repurposed materials, we were able to create a pericardiocentesis model for approximately US$247.68, which is notably cheaper than commercial models. The model is a versatile and cost-effective alternative to these more expensive options. As a standalone simulator, this model can be integrated into medical education programs that include pericardiocentesis training. However, further evaluation by medical providers who perform pericardiocentesis and subject matter experts is vital in determining the model's effectiveness and realism in the simulation training realm.
